# Structure–Function Relationships of Nanocarbon/Polymer Composites for Chemiresistive Sensing: A Review

**DOI:** 10.3390/s21093291

**Published:** 2021-05-10

**Authors:** Maryam Ehsani, Parvaneh Rahimi, Yvonne Joseph

**Affiliations:** Institute of Electronic and Sensor Materials, Faculty of Materials Science and Materials Technology, TU Bergakademie Freiberg, 09599 Freiberg, Germany; Maryam.Ehsani@doktorand.tu-freiberg.de (M.E.); Yvonne.Joseph@esm.tu-freiberg.de (Y.J.)

**Keywords:** polymer, nanocomposites, chemiresistors, electrical conductivity, mechanical properties, nanofillers, carbon nanotube, graphene, carbon black, fullerene, carbon nanoribbons

## Abstract

Composites of organic compounds and inorganic nanomaterials provide novel sensing platforms for high-performance sensor applications. The combination of the attractive functionalities of nanomaterials with polymers as an organic matrix offers promising materials with tunable electrical, mechanical, and chemisensitive properties. This review mainly focuses on nanocarbon/polymer composites as chemiresistors. We first describe the structure and properties of carbon nanofillers as reinforcement agents used in the manufacture of polymer composites and the sensing mechanism of developed nanocomposites as chemiresistors. Then, the design and synthesizing methods of polymer composites based on carbon nanofillers are discussed. The electrical conductivity, mechanical properties, and the applications of different nanocarbon/polymer composites for the detection of different analytes are reviewed. Lastly, challenges and the future vision for applications of such nanocomposites are described.

## 1. Introduction

The development of efficient chemical sensors with high sensitivity, reliability, cost effectiveness, and small size needs fundamental knowledge of chemistry and material science related with advanced sensing techniques. For chemiresistive sensors, the electrical resistance change in a sensitive layer in the presence of chemical species is monitored, which is related to the analyte concentration. There is considerable interest for using chemiresistors due to their ease of fabrication process and simplicity [1,2]. In particular, chemiresistors have been used for detection and monitoring of gases and volatile organic compounds (VOCs) in numerous applications such as vehicle and industrial emission control, environmental monitoring, household security, biomedical applications, etc. [3]. Various types of inorganic and organic materials, such as metals [4], metal oxides [5,6], conductive polymers [6], carbon nanomaterials [7], and nanocomposites [8], can be employed as chemiresistive sensitive films. Among these materials, nanocomposites material holds great promise for the development of chemiresistors due to the high adsorptive capacity, room temperature operation, variability of materials composition, ease of configuration, and low power consumption [9]. Especially, polymer nanocomposites hold significant potential for chemiresistors and have been used in electronic nose technology for more than two decades.

**Figure 1:**
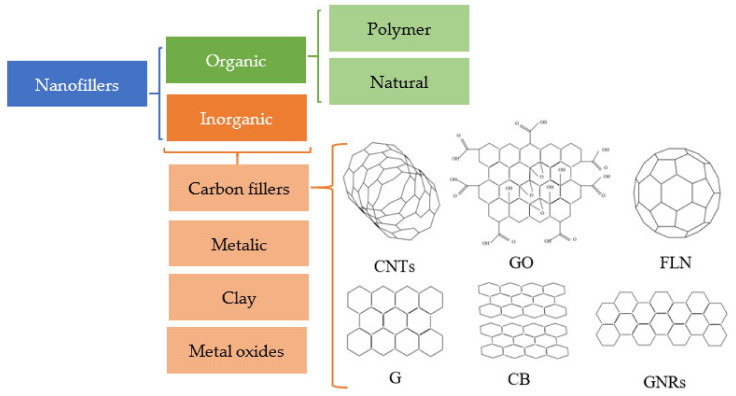
Various types of nanofillers for polymer nanocomposites.

Compared to micro- and macro-fillers, the addition of nanofillers into the polymer matrix with the same filler loading dramatically changes both the electrical and mechanical properties of polymer composites due to the high degree of contact between nanofillers and polymers, known as the “nano-effect” [10]. The control of these properties is of uttermost importance for chemiresistive performance of the sensors. On the other hand, the choice of polymer matrix, with enough functional groups providing sufficient crosslinking density and hydrogen bonding, has a strong influence on the strength and toughness of the composite as well as on sorption properties [11]. Therefore, several strategies such as the functionalization of nanofiller [12] using π–π interactions [13] and the use of multivalent cationic ions [14] have been tried to alter the crosslinking density [14,15]. It is proved that such interactions play an important role in stabilizing the spatial distribution of nanofillers in polymer matrix, supporting the improvement of mechanical properties of polymer nanocomposites. [16]. Various types of nanofillers utilized for designing and synthesizing polymer-based nanocomposites are shown in Figure 1. Among all nanofillers, carbon-based nanomaterials have recently attracted a lot of attention, and the number of investigations on polymer nanocomposites containing allotropic forms of carbon as chemiresistive sensors has increased dramatically [17,18,19,20]. Carbon is capable of forming several allotropes comprising of sp^2^- and sp^3^-hybridized carbon atoms. Among them only sp^2^–carbon allotropes, such as Graphene (G) and Graphene-derived nanofillers (e.g., Graphene oxide (GO), reduced Graphene oxide (rGO)) or Graphene nanoribbons (GNRs), carbon nanotubes (CNTs), fullerenes (FLNs), and carbon black (CB), may contribute to the electrical conductivity due to the presence of an extended π-system. These nanofillers are normally not affected by a wide variety of solvents, acids, and bases at room temperature, and they are also a cost-effective material for composite manufacturing, which exhibits a diversity of electrical and mechanical characteristics [21]. Sp^3^-hybridized carbon-based allotropes, such as nano diamonds (ND), are usually not in favor of use in chemiresistors due to their low electrical conductivity [22].

G is a two-dimensional (2D) sheet-like material with sp^2^-hybridized carbon atoms arranged in a honeycomb lattice. G and its derivatives, GO, rGO, and GNR, exhibit excellent mechanical stability and unique electrical properties. G is strong, conductive, transparent, and bendable with high mobility up to 200,000 cm^2^/Vs, low resistivity, and high carrier density, which makes it suitable for gas and vapor sensing. Moreover, a large surface area and higher interaction with gas molecules at defects when they are exposed to redox-active gas molecules leads to a change in the electrical conductivity of G and a change in free electron concentration. Recent studies show the improvement in sensing properties of gas sensors using G due to the remarkable electrical and physical properties [23]. GO consists of G having partial sp^3^–carbon atoms, surrounded with hydroxyl and epoxy functional groups, was used for the first time in 1840 [24]. The electrical conductivity of this material depends on the oxidation level. By removing the oxygen functional groups of GO and, thus, transforming sp^3^–carbon into sp^2^–carbon, rGO can be obtained with a higher π-conjugated structure, which is electrically conductive but retains defect sites making it useful for chemical sensors [25]. Actually, rGO resembles G, but with some residual oxygen and structural defects, yielding only a conductivity that is comparable to that of doped conductive polymers. GNRs are formally planar, finite, 1D cutouts of the G structure divided into two principle edge structures including armchair and zigzag structures [26]. In comparison with G as a zero-gap material, GNRs exhibit substantial energy band gaps, which is useful for tuning the electronic properties. The width, edge structure, and functionalization of GNRs make it a promising candidate for electronic devices and biomedical applications [18,27,28,29,30,31,32]. The tunable physical and electronic properties of GNRs hold promising potential for the fabrication of gas sensors [33] due to the high conductivity, resistance to electro-migration due to the strong inherent carbon–carbon bonds, extraordinary mechanical strength, and large current conduction capacity [26,33,34]. The addition of GNRs into a polymer matrix can also modify the electrical, thermal, and mechanical properties of the polymer matrix due to the interfacial properties of final nanocomposite and compatibility of this nanofiller with polymer [28,35]. CNTs are the most popular carbon-based material amongst the carbon allotropes. They are formally cylindrically wrapped G layers. CNTs are classified into two main types according to the number of wrapped G layers: single and multi-walled carbon nanotubes (SWCNTs and MWCNTs). The geometry, morphology, stability, functionality, and ease of modification of this nanomaterial makes it a suitable candidate for a variety of applications including sensors, electronics, biomedicine, renewable energy, and drug delivery [16,36,37,38,39]. FLNs are a molecular allotrope of carbon made of sp^2^-hybridized carbon atoms to form one of the spherical hollows and ellipsoid shapes. FLN molecules consist of a closed-cage made of five- and six-membered rings with 12 isolated pentagons and a different number of hexagons, depending on the size of the molecule. The FLN molecules are highly insoluble in water, only FLN-C_60_ is easily water-soluble at room temperature [40]. CB is a stacked, multilayer G and, thus, a finely particulate paracrystalline carbon with a high surface area, which is mainly used as a reinforcement agent in plastics products [41]. The introduction of CB in the polymer matrix leads to increased conductivity; therefore, it has been extensively employed for chemiresistor sensors for providing electrical signal transduction.

Actually, in order to obtain the better dispersion of filler (less aggregation), which significantly influences the conductivity and sensing properties of composite, better processability and price, it is desired to have the lowest filler contents possible. Although, CB is a suitable filler, it requires high loading for modest enhancement in properties, which causes problems in melt flow and processing due to the high viscosity of the filled materials. 

## 2. Design and Mechanism Operation of Polymer-Composite-Based Chemiresistors

To fabricate a chemiresistive sensor, a sensitive layer is deposited on the surface of interdigitated electrodes made usually from either Pt or Au. The performance of the sensor is determined by monitoring the electrical resistance changes in the sensitive layer in the presence of chemical species in which the conductivity of the sensing layer changes due to the interaction with the analyte, providing a signal related to the analyte concentration. To obtain a nanocomposite with the desired sensing properties, the compatibility of the polymer and nanofillers is an important issue, which must be taken into consideration in the design of polymer nanocomposite. The properties of polymer nanocomposites also depend on nature, type, and size as well as their electric percolation behavior, which is closely related to the amount and distribution of conductive fillers in the polymer [42]. The percolation theory evaluates the effects of internally connected nanofillers in nanocomposite on electric conductivity that decrease with decreasing filler volume fraction up to the critical value (percolation threshold, PT) [43,44]. Furthermore, to create a new material with certain functionality, the prediction of structural influence on composite behavior, fundamental understanding of physical and chemical properties, and knowledge about different interactions are required. An important parameter, which must be considered for the composite, is its matrix stiffness. It may have a direct effect on sensing performance due to analyte induced swelling of the matrix, because the contact of polymers with analyte fluids generates a volume expansion due to sorption, which mainly depends on the polymer’s solubility in the analyte [45].

**Figure 2:**
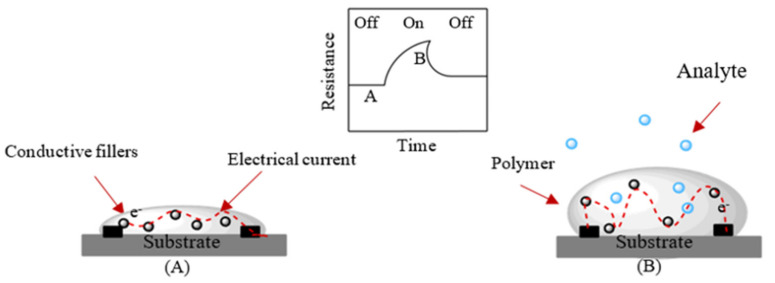
Schematic representation of chemiresistor mechanism based on swelling effect. The red dashed line gives the electrical current along the percolation pathway (**A**) before and (**B**) after swelling.

The sensing mechanism based on the swelling effect is shown in Figure 2, where the polymer nanocomposite film exposed to the target analyte and sorption of analyte molecules lead to the swelling of the polymer matrix. Therefore, the volume of the polymer increases, resulting in an increased distance between the adjacent conductive nanofillers disturbing or destroying the conductive percolation network formed by the filler in the polymer matrix, which results in the increase in electrical resistance. For effective use of the sensing mechanism, it is essential to select the right polymer–nanofiller pair and ratio as well as the preparation method. Despite the importance for the sensing mechanism, there is a strong fundamental desire for understanding the influence of swelling on electrical and mechanical properties of polymer-based composites owing to their complex microstructures. Besides the swelling effect that alters the structure of the percolation pathway, there are several other sensing mechanisms, such as electron transfer, doping–dedoping (oxidation/reduction), and protonation–deprotonation mechanisms. They are general sensing mechanisms in conducting polymers-based chemiresistive sensors, which are reviewed previously, and the readers can find more detailed information about these mechanisms [46,47,48].

## 3. Synthesis and Preparation of Nanocarbon/Polymer Composite Layers

**Figure 3:**
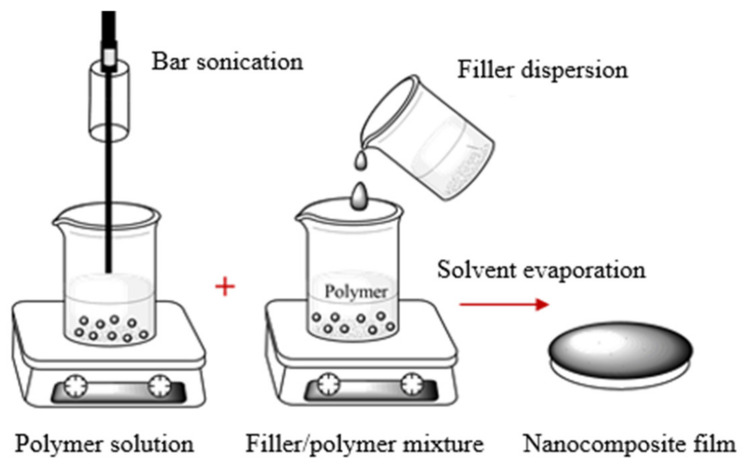
Schematic representation of solution/emulsion processing method.

Since the kind of composite preparation method influences the filler distribution in the composite, several methods were employed to provide high-quality and low-cost composite materials technology. Namely, they are in situ electro polymerization [49], solution/emulsion processing [50], vacuum-assisted self-assembly [51], in situ emulsion polymerization [20], a melt processing method [52], sol gel [53], electrochemical polymerization [54], an electro spinning method [55], melt electrospinning [56], melt mixing [57], and atom transfer radical polymerization [58]. The methods that are mostly reported for the preparation of nanocarbon/polymer composite chemiresistive layers are solution/emulsion mixing [59,60], self-assembly approaches [61], and in situ polymerization [62,63]. They are summarized below:

The solution/emulsion mixing method: Solution/emulsion mixing is commonly used for formation of nanofiller–polymer nanocomposites. Here, nanocomposites form from dispersed nanofillers in polymer solutions/emulsions. The process is followed by film casting and evaporation of solvent (Figure 3). Sometimes, surfactants are used to improve the dispersion of nanofillers in the polymer matrix and to functionalize the carbon-based particles. However, the addition of a surfactant may decrease the electrical and thermal transport properties of the nanocomposite [64]. 

**Figure 4:**
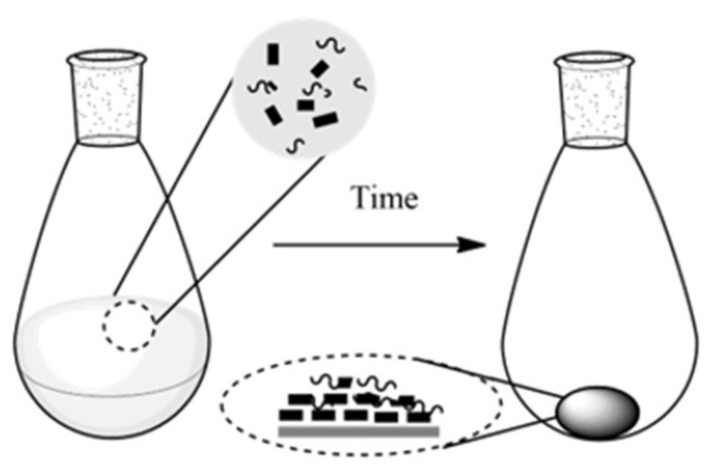
Scheme representation of synthesis of nanocarbon/polymer nanocomposite using self-assembly process.

Self-assembly: Self-assembly is a very effective method to produce nanocomposites with high loadings of nanoparticles. It organizes a specific and favorable interaction between the organic matrix and inorganic nanostructures. Nanoparticles can stack together because of strong intermolecular forces (hydrogen bonding, van der Waals bonding, π–π stacking, molecular dipole interactions) by applying a magnetic field, electric field, or current [65]. Although the fabricated polymer nanocomposites by self-assembly strategy exhibit excellent mechanical properties, they have some drawbacks such as limited material selection, setup price and complication, preparation speed, and a narrow range of interlayer polymer composition. To overcome these limitations, Putz and coworker [61] proposed vacuum-assisted self-assembly (VASA) as a facile, inexpensive, processing technique for the production of layered GO–polymer nanocomposites. The suggested method offered a facile fabrication of both hydrophobic and hydrophilic nanocomposites with the homogenous incorporation between the filler and polymers. After removal of the solvent, the nanocomposite film is obtained. Figure 4 shows the schematic of self-assembly fabrication method of nanocomposite in this research. 

**Figure 5:**
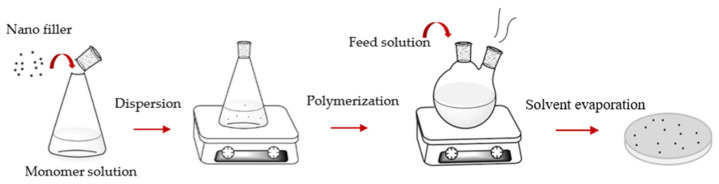
Schematic representation of in situ technique.

In situ emulsion polymerization method: In situ polymerization is one of the derivations of the emulsion polymerization technique, it uses the presence of the dispersed inorganic nanoparticles while the matrix polymerizes, thereby providing a strong interaction between the matrix and inorganic nanoparticles (Figure 5). Layered or bundled fillers may be penetrated by the monomer solution, and then, polymerization of the monomers takes place in between the interlayers/fibers to produce intercalated or exfoliated nanocomposites. This method is generally used to fabricate G-based polymer nanocomposites with conductive polymers such as poly methyl methacrylate (PMMA) [66], polyaniline (PANI) [67], polyurethane (PU) [19], etc. The process is followed by film casting and evaporation of solvent.

The in situ method has a strong influence on inorganic network formation in the polymer matrix, which makes a problem for governing inorganic material size and arrangement of inorganic domain in hybrid material [68]. In 2011, Wang’s research team [19] reported a facile and rapid preparation of a nanocomposite using a combination of G, which was derived from chemically reduced GO and PU latex by using the in situ polymerization technique. They could improve the tensile strength and storage modulus of the PU by 239 and 202%, respectively, just with the incorporation of 2.0 wt.% of G. To obtain colloidally stable hybrid latexes for possible application as electrically conductive coatings, Arzac et al. [20] synthesized the water-born rGO/polymer nanocomposites by emulsion mixing and in situ polymerization. The polymer composed of poly (methyl metarcylate/butyl acrylate) was joined to rGO stable dispersion prepared by the reduction in GO in the presence of polyvinylpyrrolidone (PVP). The establishment of covalent bonds between polymers and rGO by the in situ polymerization technique offered an excellent way to produce the composite. The developed in situ composites showed decreased aggregation of rGO, uniformly distributed rGO in the polymeric matrix, and high colloidal stability. 

Among these methods, the nanocomposite prepared by the in situ polymerization technique shows effective dispersion of the nanofiller in the polymer matrix; however, the procedure is complex, and it requires expensive reactants [69]. In the self-assembly approach, the molecules are used for building up the complex molecular structure under environmentally friendly conditions. This method can control the composition of composites; however, achieving a high quantity of material is not easy and mostly expensive [70,71,72]. By using the solution mixing method, the sample size can be increased. This technique possesses the dispersion of nanofiller with low viscosity condition, which makes it an effective technique for synthesizing composites with uniformly dispersed nanofillers [73].

## 4. Nanocarbon/Polymer Composite as Chemiresistive Sensors

### 4.1. G;GO;rGO/Polymer Composite

**Figure 6:**
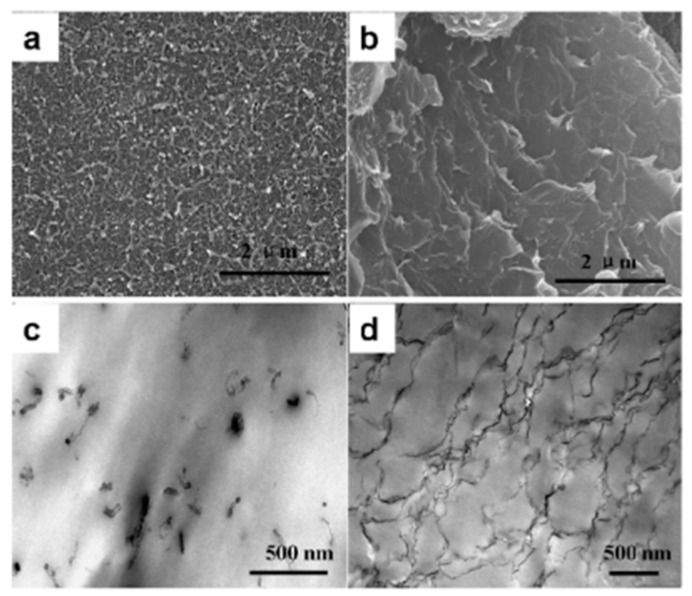
SEM images of PS nanocomposites with (**a**) 1.5 wt.% CNT and (**b**) 1.5 wt.% G. TEM images of PS nanocomposites with (**c**) 1.5 wt.% CNT and (**d**) 1.5 wt.% G [80], reprinted with permission from American Chemical Society.

G-derivatives/polymer-based composites have attracted great attention for the fabrication of chemiresistor sensors due to their high sensitivity, long reliability, and low power consumption [74]. They have been synthesized using various methods with superior electrical and mechanical properties compared to pure polymers, which have been reviewed by Lawal and Alshamaileh [75]. As mentioned before, the preparation and dispersion method of nanofiller into polymer matrices influences the properties of polymer nanocomposite. In this regard, the effect of rGO incorporation methods into PMMA on the electrical and mechanical properties of nanocomposite was investigated by Tripathi and coworkers [66]. They prepared the composites by using different synthesizing methods and various amounts of rGO (0.1–2% *w*/*w*). The results showed the dependency of the electrical conductivity of composites on the rGO loading range and the methods of incorporation into the polymer. The percolation threshold concentration was dependent on the quality of the filler dispersion and agglomeration. To evaluate the dispersion quality of rGO into polymer, the morphology of the prepared composites was investigated using scanning electron microscopy (SEM) and high-resolution transmission electron microscopy (HRTEM). According to the recorded SEM and HRTEM images, the dispersion of rGO into the polymer was better in the prepared samples using the casting method, while the composite prepared by in situ polymerization indicated nonuniform dispersion of rGO into the polymer. With the incorporation of 2 wt.% rGO in the PMMA composite using the in situ polymerization of MMA in the presence of rGO and PMMA beads, the electrical conductivity increased by a factor of 10^7^, while the composite synthesized by the casting method showed an increase of 10^8^ times in electrical conductivity. The addition of rGO into the polymer demonstrated a significant effect on the mechanical properties of composites and improved the modulus and stiffness of the composite. However, the strength of the composite prepared by in situ polymerization decreased with the addition of 1 wt.% rGO compared to the neat polymer (~24 MPa) due to the weak dispersion of rGO within the polymer and presence of agglomeration. In comparison, the composite containing 2 wt.% rGO prepared with the casting method showed the same strength as 1 wt.% composite prepared by the in situ/PMMA bead method due to the better dispersion and good interaction of filler and the polymer matrix. Although in situ thermal reduction in GO is a simple and green approach in the fabrication of G-based polymer composites, it lacks efficiency due to the limitation of the thermal reduction temperature. To overcome this limitation, Xu and coworkers [76] synthesized the rGO/polycarbonate (PC) nanocomposites via the solvent exchange method, followed by high-temperature in situ thermal reduction. SEM and TEM images of the nanocomposites homogenously exhibited dispersion and full exfoliation of rGO sheets. It could be attributed to the used solvent exchange and the in situ thermal reduction method, which avoid the aggregation of GO during its reduction. As a result, the nanocomposites exhibited a low percolation threshold of ∼0.21 vol.% and a conductivity of 0.041 S × m^−1^ at an rGO content of 1.09 vol.%. In order to investigate the influence of the nanofiller content, Yang et al. [77] prepared the rGO/poly (vinyl alcohol) (PVA) nanocomposite using a reduction solution process with different rGO content. The good dispersity and formation of a network nanostructure of rGO in PVA were confirmed by TEM. Actually, the functional groups of GO and rGO are the reason for their good dispersibility and high compatibility with water-swelling polymers such as PVA. The electrical conductivity of rGO/PVA increased from 6.04 × 10^−3^ to 5.92 S × m^−1^ with the addition of rGO content, from 4 up to 14 wt.%, which is due to the formation of the rGO network and interconnection structure of rGO. In other research, the self-assembly method was used for the preparation of a rGO/polystyrene (PS) composite by dispersion of rGO in a different loading ratio (0.2–0.9 vol.%) [78]. First, PS microspheres were covered with GO via self-assembly, and then, GO was reduced. Finally, composite film was prepared by hot pressing. The morphological investigation demonstrated that the rGO sheets tend to aggregate together before the hot-pressing process. However, at the temperature above the softening point of PS, polymer forms a coherent film and GO sheets cannot interpenetrate the PS, remain as a network in the composite, and form the conductive pathways in PS matrix, which greatly enhance the electrical conductivity. In other words, polymer latex avoided the aggregation of rGO and led an improvement of good dispersion of rGO in the polymer, which was confirmed by TEM images. The electrical conductivity was improved with rising rGO loading range up to 0.9 vol.% and a low percolation threshold of 0.2 vol.%. Another important factor to increase the mechanical properties and electrical conductivity of G-derivatives/polymer composites is surface functionalization and modification of G, which improves their homogeneous dispersion into the polymer matrix and interfacial interaction. GO and rGO with many oxygen functional groups are more popular than pristine G, because they can be easily homogeneously dispersed in different polymer matrices. However, in order to achieve better dispersion, several surface modifications of G, GO, and rGO have been done [75]. In this regard, Park and his coworker [79] synthesized two different polyimide (PI) composites using rGO and reduced iodo phenyl functionalized GO (r-I-Ph-GO) with various content (0.5, 1, and 2 wt.%) by using an effective method of in situ polymerization. They studied the dispersion of rGO and r-I-Ph-GO in water using UV/vis spectroscopy and observed the higher dispersibility of r-I-Ph-GO rather than rGO due to the iodo groups, which improve the interaction of rGO with water. The better reinforcement effect was observed for the r-I-Ph-GO/PI composite compared to rGO/PI, resulting in better electrical and mechanical properties. The electrical conductivity of 5.2 × 10^-2^ S × m^−1^ was achieved by the addition of 1 wt.% r-I-Ph-GO within PI, which is 10^7^ times more than the electrical conductivity of the rGO/PI composite. The reduction efficiency of a composite with I-Ph-GO is higher than the composite containing rGO due to inducing deoxygenation of the iodo group on I-Ph-GO during the imidization process and catalyst effects. Young’s modulus and tensile strength of PI matrix improved from 2.5 to 7.9 GPa and 75.7 to 111 MPa, respectively, for the r-I-Ph-GO/PI composite. It has also been reported that G-derivatives/polymer composites show much better mechanical and electrical properties than other carbon filler-based polymer composites. Qi et al. [80] could enhance the electrical conductivity and percolation threshold of a composite based on G/PS with the addition of ultra-low G content. They synthesized both G/PS and CNTs/PS composites using the solution mixing method and compared the changes in electrical conductivity and mechanical properties of the composites. A significant improvement was observed in the mechanical and electrical conductivity of G-reinforced PS rather than CNT-reinforced PS due to the great interfacial contact area in G and the formation of a pseudosolid-like network. SEM and TEM analysis revealed that G-sheets due to π–π interactions were homogeneously dispersed in the PS matrix (Figure 6b,d), while CNT agglomerated and, compared to G, did not uniformly disperse in the PS matrix (Figure 6a,c).

In another report, Liu et al. [81] using the solution mixing method fabricated a composite of PS and ionic liquid-functionalized G with high structural homogeneity and excellent conductivity of 13.84 S·m^−1^, which was 3–15 times higher than the values reported for SWCNTs-filled PS composites [82]. The high electrical conductivity of fabricated G/PS compared to SWCNTs/PS can be attributed to the layer structure of G with high aspect ratio and without the need for helicity control, which provides its relatively easily modification with desirable chemical and physical properties for simple incorporation into different polymer matrices. However, the combination of G-derivatives with CNTs can be used as hybrid reinforcement nanofillers, which show synergistic effects and improve the mechanical and electrical characteristics of nanocomposites. To this aim, Li et al. [83] prepared the PVA nanocomposites using GO and CNTs as a hybrid nanofiller with various weight ratios and compared the properties of the GO–CNTs/PVA composites with CNTs/PVA and GO/PVA composites. In the presence of GO, CNTs tend to be wrapped by GO to form a nanoscroll structure rather than self-assembling into bundles, which was confirmed by FESEM images. In addition, the optical images of different CNTs/PVA, GO/PVA, and GO–CNTs/PVA composites indicated that the addition of GO improves the dispersion of CNT significantly, resulting in the formation of a uniformly black color GO–CNTs/PVA composite. Due to the high dispersity of GO in water and the strong affinity between GO sheets and CNTs, the hydrophobic surface of the CNT is covered by GO, which improves the dispersion of CNTs in the PVA matrix. The fabricated GO–CNT/PVA composite with GO–CNT content between 2–3 wt.% showed synergistic effects with superior mechanical properties than those of single GO- or CNT-enhanced PVA composite films. Table 1 presents a summary of the electrical conductivity and mechanical properties of different G-derivatives/polymer composites.

The chemiresistive sensing behavior of G, GO, and rGO-filled polymers toward gas and vapor molecules is indicated in Table 2. G, GO, and rGO-filled conducting polymers demonstrated enhancement in sensitivity and selectivity of the chemiresistive sensor towards different gas and vapor analytes compared with a pure G-derivatives- or polymer- based sensor. Li et al. [67] developed a highly sensitive sensor based on a GO/rambutan-like polyaniline hollow hybrid (PANIH) composite assembled on the polyethylene terephthalate (PET) substrate for detection of NH_3_ at room temperature for the addition of 0.5 wt.% of GO into the PANI matrix. They fabricated the GO/PANIH composite via the in situ chemical oxidation polymerization method and loaded it on a flexible PET. FESEM and TEM analysis showed that PANIH were uniformly grown and bonded on the surface of GO, which was additionally verified by X-ray powder diffraction measurement. The highest sensor response was obtained toward NH_3_ in a linear range of 31.8–100 ppm with lower limit of detection (LOD) (50 ppb) compared to the reported chemiresistive sensor based on MWCNTs/PANI [84]. The response mechanism is protonation/deprotonation of PANIH upon exposure to NH_3_. The prepared PANI by in situ chemical oxidation polymerization under acidic condition is recognized as a conductive form of emeraldine salt, which has an abundance of protons. With the exposure of protonated PANI (emeraldine salt) to NH_3_, the protons are transferred to NH_3_ molecules to form ammonium ions and cause a reduction in PANI from conductive emeraldine salt state to nonconductive intrinsic emeraldine base state and increasing resistance. In addition, rGO provided a larger specific surface area for PANIH, which caused more adsorption of NH_3_ and improvement of sensing properties. Therefore, the synergistic effect of GO filler and PANIH led the high sensing behavior of the designed sensor. A flexible NH_3_ sensor was made based on a nanocomposite of G and poly (3,4-ethylenedioxythiophene): poly(styrenesulfonate) (PEDOT: PSS) by a simple and low-cost inkjet-printing technique on a transparency substrate with prefabricated electrodes. The printed gas sensor showed high sensitivity and selectivity to NH_3_ in a concentration range of 25–1000 ppm. The sensing response was clarified based on three mechanisms including direct charge transfer between NH_3_ molecules and G/PEDOT: PSS composite, a reduced reaction between chemisorbed oxygen and NH_3_, and a swelling effect through the diffusion of NH_3_ molecules into the polymer composite. The high performance of the proposed sensor could be attributed to the increased specific surface area by G and superior interactions between the G/PEDOT: PSS composite and NH_3_ molecules via the π electrons network [85]. Jang and coworkers [86] prepared reproducible and rapid NH_3_ gas sensors using nanocomposites of polypyrrole (PPy) with GO and rGO. FESEM analysis indicated that the rGO/PPy nanocomposite compared to the GO/PPy nanocomposite shows a better and uniform morphology and good dispersion of rGO, which is important for the sensing performance of a nanocomposite. As expected, rGO/PPy nanocomposite indicated the obviously higher gas sensitivity in terms of electrical resistance changes towards NH_3_ compared to the GO/PPy nanocomposite due to the improved electrical conductivity and higher available surface for gas sensing. The sensing mechanism was based on the charge transferring between PPy and NH_3_ as electron-donating molecules, which decrease the doping level of PPy and the reduced number of carriers, thus leading to a variation in the electrical resistance of PPy. The resulting electrical resistance change was transferred to the electrode through the dispersed GO and rGO. The sensitivity of rGO/PPy increased from 0 to 7% during the initial 25 min NH_3_ exposure, and LOD has not been reported in this paper. 

**Figure 7:**
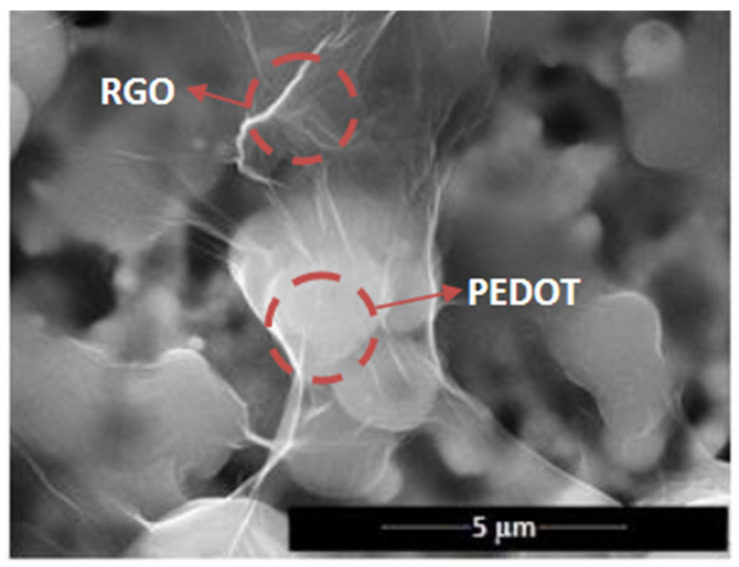
SEM images of rGO (RGO)/PEDOT film and conduction pathway [92], reprinted with permission from Elsevier.

Wu et al. [87] fabricated a NH_3_ chemiresistor sensor based on the G/PANI nanocomposites with a wide linear response range from 1 to 6400 ppm. As compared to PANI film, the G/PANI nanocomposite exhibited a much higher sensitivity (ca. 5 times), faster response, excellent reproducibility, and lower LOD (~1 ppm) than that of PANI (~10 ppm) for NH_3_ gas. The sensing performance has not been fully understood and explained with different mechanisms including the de-doping of PANI via the transfer of protons on –NH–groups of PANI to NH_3_, charge transfer from NH_3_ to PANI, and also the swelling effect. GO/PANI composites-based chemiresistors have not only been used for the detection of polluted gases, but also for alcohol sensing applications. GO/PANI composites were prepared using the polymerization of aniline monomer in the presence of GO under acidic conditions [88]. The final composite with enhanced electrical conductivity to 241 S·m^−1^ was subjected to the detection of methanol, ethanol, and propanol vapors. The results revealed the high sensitivity (∆R/R0 = 20.9−37) of GO/PANI toward methanol vapor (100–500 ppm), which is due to the strong hydrogen bonding between methanol and the polymer chain confirmed with Fourier transform infrared spectroscopy (FTIR) and density functional theory studies. To evaluate the selectivity and sensitivity of GO/PANI towards methanol, the sensor response was investigated for the detection of ethanol and propanol. Due to the low polarity nature of ethanol and propanol compared to the methanol, fewer sensitivities [∆R/R0 = 3.77 and 3.10] were recorded. As mentioned before, a conducting polymer, especially the nanostructured conducting polymer, plays important roles in the development of the G-based sensing platforms. In this regard, a NO_2_ chemiresistor sensor based on rGO and the porous conducting polymer PEDOT nanostructure was developed [89]. The porous PEDOT nanostructure was deposited on the surface of rGO sheets by using a fast-thermal treatment during the in situ polymerization of 3,4-ethylenedioxythiophene (EDOT) monomer. The high surface area and porous nanostructure of the developed nanocomposite improved NO_2_ adsorption and desorption. The proposed sensing device based on rGO/PEDOT showed a fast response, selectivity, and sensing performance over a wide linear range of NO_2_ concentration (500 ppb to 20 ppm) due to the excellent synergetic effect between rGO sheets and porous PEDOT. Exfoliated G blended with PMMA was used as a sensing material for the development of chemiresistor sensors for the determination of formaldehyde at room temperature [90]. To prepare a sensitive and selective sensor, the ratio of G/PMMA was optimized (7:2 mg) and tested for methanol, acetone, and tetrahydrofuran, other than formaldehyde. It was demonstrated that the ratio of G/PMMA has an important role in the sensing performance of a composite. The optimized sensor could selectively detect formaldehyde in a relatively wide range of concentration from 0.05 to 5.0 ppm with LOD of 0.01 ppm. The electrical response of the sensor was explained by two different mechanisms: the swelling of the polymer by the absorption of formaldehyde vapor, which raises the volume of polymer and increases the electrical resistance by increasing the distance between G flakes, and the formation of conductive pathways inside the G/PMMA composite by the quantum tunneling effect. Tung et al. [91] synthesized a nanocomposite based on polymerized ionic liquid (PIL)-modified rGO and PEDOT and used it as chemiresistive sensor to effectively detect trace levels of different VOCs including tetrahydrofuran, chloroform, benzene, and methanol. The sensing mechanism was explained by van der Waals interactions between organic vapors and the composite. The sensor provided well-defined signals with high reproducibility and reversibility towards the analyte vapors over the concentration of 1–90 ppm. In another study, Dunst and her team [92] prepared a NO_2_ sensor based on a rGO/PEDOT nanocomposite made by a fully electrochemical route. rGO/PEDOT nanocomposite film was deposited by electrochemical polymerization and reduction on the surface of interdigitated electrodes and used as a sensing layer for the detection of NO_2_ in a concentration range of 5–100 ppm. The SEM images shown in Figure 7 revealed that the rGO sheets homogeneously covered the PEDOT polymer during the electropolymerization, which forms the electric conduction pathways for electrochemical sensing.

Table 2 shows the results at 80°C due to the synergetic effect between PEDOT and rGO sheets. The sensing mechanism was assumed based on electron transferring between NO_2_ gas and rGO/PEDOT composite. After the adsorption of NO_2_ gas as a strong electron acceptor on the surface of the composite, electrons transfer from the rGO to NO_2_ molecules and improve the electrical conductivity by increasing the hole concentration in the rGO sheet. However, at room temperature, the response to humidity was much higher, which could compromise sensor reliability. A flexible and portable chemiresistor-based G-cellulose nanocomposite test paper (NCTP) comprising a G-lamellar membrane on a polymer substrate was introduced by Jiang’s team for rapid liquid recognition [93]. In the proposed sensing platform, the liquid droplet would penetrate into NCTP, impact the contact state and the carrier density of the G-sheet, and alter the total resistance. The proposed NCTP was successfully tested for the qualitative and quantitative analysis of various liquids, including water, number of organic solvents, and metal salts. Two sensing possible mechanisms including the swelling effect and the formation of hydrogen bonding between the polar solvent and the NCTP were proposed for the fabricated NCTP-based sensor. Such a carbon-based polymer nanocomposite as a chemiresistor may have promising applications in versatile, flexible, and portable liquid-sensing devices for inactive healthcare, mobile beverage quality testing, and environmental monitoring.

### 4.2. GNRs/Polymer Composite

The incorporation of GNRs with exceptional properties, large surface area, and rich-edge chemistry into the polymer latex causes a significant improvement in load transfer effectiveness and electrical, thermal, and mechanical properties of polymers composites [35]. Rafiee et al. [28] investigated for the first time the influence of GNRs on enhancement in load transfer effectiveness and mechanical properties of the polymer nanocomposites. They could obtain a 30% increase in Young’s modulus, and a 22% increase in tensile strength of the composite by the addition of ~0.3 wt.% nanoribbons into the epoxy. In another work, Shang et al. [29] fabricated a GNRs/PVA nanocomposite using a solution mixing method. The SEM images indicated that GNRs with a ribbon sheet shape were homogeneously dispersed in the PVA matrix, which may be attributed to the formation of hydrogen bonding between oxygen groups of GNRs and hydroxyl groups of the PVA. They observed a significant increase in the mechanical performance of PVA/GNRs nanocomposite with 2.0 wt.% GNRs loading. Tensile strength of PVA/GNRs nanocomposite was increased by 85.7% from 18.2 to 33.8 MPa and Young’s modulus was enhanced by 65.2% from 0.070 to 1.164 GPa compared to neat PVA. Li et al. [94] reported a nanocomposite of PANI and GNRs by the in situ polymerization of aniline in the presence of GNRs and used it for the development of capacitive pseudocapacitors. GNRs served as substrate for PANI growth, and according to the recorded SEM and TEM images, PANI was grown on and around the GNRs, covered the external surface of the GNRs, and formed one-dimensional ordered wires. The synergistic combination of electrically conductive GNRs and highly capacitive PANI made PANI/GNRs a superb electrode material with highly improved electrical conductivity and mechanical properties for long-lived energy storage devices.

**Figure 8:**
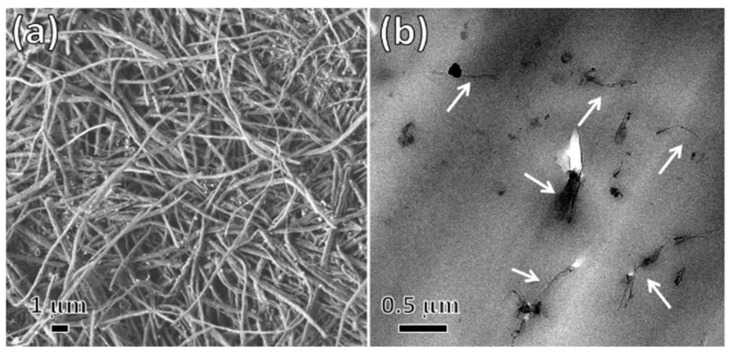
(**a**) SEM images of agglomerated edge-functionalized GNR and (**b**) TEM image of nanocomposite loaded by 0.15 wt.% edge-functionalized GNRs (white arrows mark the individually dispersed GNRs) [95], reprinted with permission from Elsevier.

It has been proved that the edge functionalization of GNRs improve the interfacial properties and compatibility in the polymer/GNRs nanocomposite. Nadiv and coworkers [95] functionalized the edges of GNR first with polyvinylamine (PVAM) chains and then incorporated it into a brittle epoxy polymer matrix. The morphological investigation based on SEM (Figure 8a) and TEM (Figure 8b) images showed that the edge-functionalized GNRs were better dispersed in the polymer matrix. They showed that the edge functionalization plays a crucial role in achieving genuine matrix reinforcement, mainly indicating superior mechanical properties as compared to the pristine GNR/polymer nanocomposite. These results are attributed to the compatibility of the PVAM groups, which participate in the polymerization process of the epoxy matrix and improve the interfacial adhesion and the subsequent stress transfer between the matrix and the filler.

Table 3 presents a summary of the electrical conductivity and mechanical properties of GNRs/polymer composites.

The presence of oxygen functional groups of GNRs facilitate rapid electron transfer that make the related nanocomposite suitable for sensing applications [30,35]. Despite the outstanding potential of a GNRs/polymer nanocomposite, there has been very limited research on the development of a GNRs/polymer nanocomposite in sensor applications. Trajcheva et al. [30] fabricated the nanocomposite of GNRs/poly (methyl methacrylate-butyl acrylate (Hydroxyethyl)methacrylate) (p(MMA-BA-HEMA)) in different GNRs loading ranges (0.2–3.0 wt.%) by in situ mini-emulsion polymerization and used them as sensors for the detection of CO, NH_3_, and N_2_O gases. The prepared nanocomposite with 3.0 wt.% of GNRs demonstrated excellent mechanical properties with up to 66-fold, nine-fold, and a 2 orders of magnitude increase in Young’s modulus, offset yield stress, and storage modulus, respectively. The designed sensors based on the GNRs/p(MMA/BA/HEMA) nanocomposite with 3.0 wt.% of GNRs loading could successfully detect CO, NH_3_, and N_2_O in a concentration range of 70–1000 ppm, at room temperature, in a short time with very good reproducibility. Moreover, the sensors showed higher sensitivity and selectivity towards NH_3_ rather than N_2_O and CO samples. This could be attributed to the interaction of NH_3_ through hydrogen bonding with GNRs/p(MMA/BA/HEMA) nanocomposite; whereas, N_2_O and CO interact exclusively by van der Waals interactions.

### 4.3. CNT/Polymer Composite

**Figure 9:**
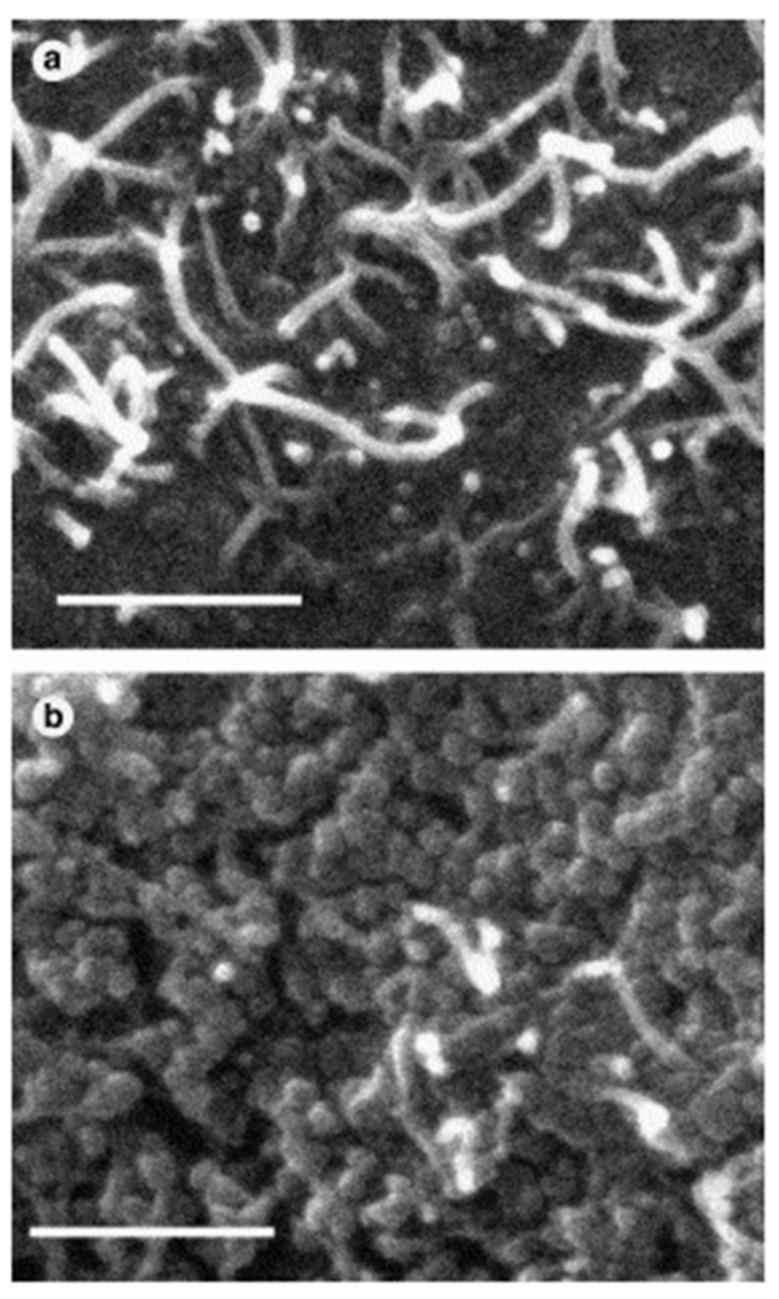
SEM micrographs (**a**) 0.5 wt.% SWCNT/iPP and (**b**) 1.0 wt.% SWCNT/iPP composites [100], reprinted with permission from Elsevier.

Due to the extraordinary properties of CNTs, a lot of research work has been done to explore the enormous potential of these nanofillers and evaluate the related CNT/polymer composites [96,97]. Incorporating only small amounts of these nanofillers into the polymer matrix can provide remarkable mechanical and electrical properties for a final composite [96,97,98]. Bokobza [99] proposed a composite of styrene–butadiene rubber (SBR) filled with 10 wt.% MWCNTs using the solution mixing method. The relatively homogeneous dispersion of MWCNTs in SBR was investigated by TEM and atomic force microscopy (AFM) analysis; however, some bundles or agglomerations of MWCNTs were also observed. To evaluate the mechanical and electrical properties of MWCNT/SBR, he prepared a CB/SBR composite and made a comparison. The addition of 10 wt.% MWCNTs to SBR enhanced the electrical conductivity and caused a 470% increase in modulus, while the stress and strain at rupture increased by 670 and 47%, respectively, which was higher than those provided by a same amount of CB. Furthermore, he reported that the addition of CB to SBR/MWCNT improves the formation of connected filler structures, which leads to a reduction in the percolation threshold (between 2 and 3 wt.%) and an improvement in the electrical properties. Lopez Manchado and coworkers [100] studied the mechanical properties of a SWCNT/isotactic polypropylene (iPP) composite, which was synthesized using the solution mixing method and then compared with the mechanical properties of composites containing CB as a filler. The morphological characterization based on SEM images (Figure 9) showed that the SWCNTs at low concentration (0.5 wt.%, Figure 9a) can homogeneously disperse in iPP matrix, while at the higher concentration (1.0 wt.%, Figure 9b), they tend to aggregate as the bundles. As expected, they observed that low concentrations of SWCNTs (less than 1 wt.%) resulted in an increase in Young’s modulus and tensile strength, which were noticeably higher than those obtained for CB/iPP composites. According to the achieved results, with the incorporation of 0.75 wt.% SWCNT, Young’s modulus and tensile strength considerably enhanced due to strong interfacial bonding with respect to the un-reinforced polymer. 

In another study, Mazinani et al. [101] prepared CNT/PS nanocomposites through electrospinning of PS/di-methyl formamide solution containing various concentrations and types of CNTs. They used styrene-butadiene-styrene type as a copolymer and an interfacial agent to modify the dispersion of CNTs in PS solution before electrospinning. The dispersion of CNTs in PS, the morphological characteristics of nanocomposite, and the effect of copolymer addition to the final CNT dispersion inside PS were studied by optical microscopy analysis. As they observed the addition of more MWCNTs (from 1–5%), it became difficult to disperse the MWCNTs in PS, and more agglomerates were formed. The addition of copolymer reduced the size and amount of agglomerations and improved the dispersion of MWCNTs in the polymer matrix. The best electrical conductivity was obtained at the percolation threshold of 5% MWCNT. At the higher concentrations of CNT, electrical conductivity was decreased, which could be related to the coating of CNTs with copolymer. Moreover, they fabricated the nanocomposites with 5% SWCNT and a double-wall carbon nanotube (DWCNT). Despite poor dispersion, the best electrical conductivity was obtained in SWCNT/PS nanocomposite (0.037 S/m) compared to MWCNT/PS (0.0053 S/m) and DWCNT/PS (0.005 S/m) nanocomposites. In contrast with electrical conductivity results, DWCNT/PS exhibited the best mechanical properties, which might originate from the small size of DWCNT compared to MWCNT and more interface and connection with matrix, and it is easier to disperse compared to SWCNT. To stabilize the dispersion and prevent the aggregation of SWCNT as nanofiller, Grunlan et al. [102] firstly stabilized SWCNTs using Gum Arabic as an effective stabilizing agent for SWCNTs and then combined them with PVAc emulsion to create an electrically conductive composite. They could reach a percolation threshold below 0.04 wt.% SWCNT.

It is widely recognized that the surface modifications or the functionalization of CNTs promotes the dispersion stability and leads to the coupling of CNT with the polymeric matrix and fabrication of high-performance CNT/polymer composites [97]. To this aim, Sen et al. [103] fabricated two different SWCNT/PU composites using the electrospinning technique with pristine SWCNTs and ester (EST)-functionalized SWCNTs to demonstrate the effect of the functionalization of SWCNTs on the mechanical properties of SWCNT-reinforced composites. The EST-SWCNT/PU composite exhibited better mechanical properties than those fabricated with pristine SWCNT/PU. The tensile strength of the EST-SWCNT/PU composite enhanced by 104% from 7.02 to 14.32 MPa, while an increase of only 46% (from 7.02 to 10.26 MPa) was achieved by a pristine SWCNT/PU composite compared to pure PU. This improvement may be attributed to the enhanced interaction between SWCNTs and the polymer matrix due to the attached long chain on ester-functionalized SWCNTs. In addition, the polar groups in the ester functionality provide opportunities for the formation of hydrogen bond interactions with the polymer and amidation reactions with free amines in PU. Skákalová et al. [104] reported that SWCNTs treated with thionyl chloride (SOCl_2_) show an increasing electrical conductivity by a factor of 5 due to the doping effect of SOCl_2_. They synthesized nanocomposites based on PMMA and 10 wt.% pristine SWCNTs and SOCl_2_-treated SWCNTs and could obtain maximum conductivities of 10^4^ S·m^−1^ for SOCl_2_ treated SWCNTs/PMMA compared to SWCNTs/PMMA with a conductivity of 1700 S·m^−1^. Such an electrical conductivity improvement was explained by the stronger interaction of SWCNTs with polymer due to the ionic doping of SWCNTs. Later, nanocomposites of SOCl_2_-functionalized SWCNTs/PMMA with different SOCl_2_ functionalized SWCNTs concentrations (0.1–0.5 wt.%) were prepared by the solution mixing method. Compared with the neat PMMA (with conductivity of 10^−13^ S·m^−1^), the electrical conductivities of 0.1 and 0.5 wt.% SOCl_2_-functionalized SWCNTs/PMMA composites were considerably enhanced to 0.035 and 47 S·m^−1^, respectively [105]. To improve the homogeneous dispersion of MWCNTs in the poly (vinyl chloride) (PVC) matrix and the efficiency of load transfer from the matrix to the MWCNTs, Shi et al. [106] grafted poly (n-butyl methacrylate) (PBMA) onto MWCNTs using atom transfer radical polymerization. TEM imaging of the composite confirmed the covering of MWCNTs with PBMA. The prepared nanocomposite with 0.2 wt.% of PBMA-grafted MWCNTs indicated significant increases in Young’s modulus, yield stress, and tensile strength by 40, 74, and 84%, respectively. The miscibility between PVC and PBMA facilitated the homogeneous dispersion of MWCNTs in the PVC matrix and led to an effective load transfer between the polymer matrix and nanotubes.

Deng et al. [107] studied the effect of coated MWCNTs with high-density polyethylene (HDPE) on the mechanical properties of HDPE-MWCNTs/PP composite produced by melt mixing. According to the TEM images, the quality of dispersion and orientation of MWCNTs in the PP matrix with the inter-phasing of HDPE between CNTs and matrix was improved. A considerable improvement from 1.4 to 1.8 GPa for Young’s modulus and an increase from 34 to 38 MPa for yield strength were obtained at low loadings (0.5 wt.%) of HDPE-MWNCTs. These reasonable results could be related to the HDPE coating, which improves the dispersion and stress transfer of the MWCNTs with the host matrix. The electrical and mechanical properties of different CNT/polymer-based nanocomposites are given in Table 4.

As seen, CNT is an ideal filler for fabricating polymer composites and has great potential in altering the electrical and mechanical properties of polymer matrices. However, as reviewed, the quality and properties of CNT/polymer nanocomposites depends on many factors, such as type of CNTs, uniform dispersion, loading concentration, preparation method, surface functionalizing, and/or modification.

With their excellent range of properties, CNT/polymer composites can be utilized as multifunctional materials for sensing applications. The sensing performance such as sensitivity and LOD for chemiresistive sensors based on CNT/polymer composites is presented in Table 5. He et al. [108] prepared a MWCNTs/PANI composite using the in situ polymerization method and used it as a sensor for the detection of NH_3_. They also investigated the relationship between the thickness of PANI coatings and the gas sensing properties of NH_3_. The MWCNTs/33 wt.% PANI composite showed high sensitivity, with relatively faster sensor response and recovery. The MWCNTs/33 wt.% PANI sensor exhibited a linear response to NH_3_ in the range of 0.2–15 ppm with a response time of about 10 to 120 s, which varied with the concentrations of NH_3_. The sensing characteristics of the MWCNTs/PANI composite to NH_3_ can be related to the combined effect of doping/de-doping of PANI and the electron transfer between the NH_3_ molecules and MWCNTs. Later, Abdulla et al. [84] reported a gas sensor for the detection of NH_3_ based on carboxylated MWCNTs (C-MWCNTs)/PANI composite prepared using the in situ oxidative polymerization method and could considerably reduce the sensor response time compared to the pristine MWCNTs/PANI composite [108]. Actually, the modification of MWCNTs with carboxylate groups improved the dispersion of MWCNTs in aniline during the polymerization process and led to uniformly covering of MWCNTs by PANI, which was confirmed by TEM investigations. The gas sensor properties of the C-MWCNTs/PANI nanocomposite towards NH_3_ at trace level concentrations (2–10 ppm) were analyzed, and its performance was compared with a C-MWCNTs-based sensor. The C-MWCNTs/PANI nanocomposite showed a very fast response (6–24 s) and good reversibility (35–62 s) compared to the C-MWCNT (more than 1000 s), which they attributed to the enhanced charge transfer through the polymer layer on C-MWCNT. Sensor response for C-MWCNTs and the C-MWCNTs/PANI nanocomposite for various NH_3_ concentrations was found to be 2.58–7.2 and 15.5–32%, respectively. The sensing performance upon exposure of NH_3_ molecules was based on the change in electrical properties based on charge transferring between MWCNTs and gas molecules. Sharma’s team [109] studied two types of MWCNTs/conducting polymer composites using PEDOT: PSS and PANI to compare their gas sensing properties towards NH_3_. The PEDOT: PSS polymer composite compared to PANI was found to be more sensitive (with a sensitivity of ~16%) with less response time (~15 min). Since sensor recovery was difficult at room temperature, they proposed a new approach based on the combination of heat and DC electric field that could desorb chemisorbed NH_3_ from a CNT surface completely and reduce the recovery time from 48 h to 20 min. To improve the sensing performance of the PANI/CNTs nanocomposite, two kinds of hierarchical p-PANI/CNT and n-PANI/CNT fibers were prepared [110]. Both samples showed higher sensitivity, better reversibility, and faster response and recovery time than the reported PANI/MWCNTs composites due to the one-dimensional morphology, hierarchical structures, the enhanced carrier mobility, and p–n heterojunctions. Compared to other proposed nanocomposite-based sensors [84,108,109], very low LOD of 19.6 and 6.5 ppb for NO_2_ and NH_3_ was observed, respectively. The response times of p-PANI/CNT and n-PANI/CNT to 50 ppm of NO_2_ and NH_3_ were reported as 5.2 and 1.8 s, respectively, indicating the real-time response. Li and coworkers [111] developed an MWCNTs/PANI composite with 25 wt.% MWCNT using the in situ polymerization method and used it as a chemiresistive sensor for the detection of aromatic hydrocarbon vapors including benzene, toluene, p-xylene, m-xylene, o-xylene, and ethylbenzene. The proposed sensor exhibited a response in the order of polarity of the molecules upon varying the vapor concentration from 200 to 1000 ppm. Although the interaction between PANI and MWCNTs increased the conductivity of nanocomposite, it reduced the magnitude of sensor response due to the interaction of MWCNTs with aromatic molecules, because PANI has an interchain distance of 0.9 nm that should be accessible for small-size aromatic molecules (0.7 nm). The recovery of the composite was very poor at room temperature, which might be related to the presence of intra-benzene molecules within CNTs. The gas-sensing performance of functionalized (F)-MWCNT/PMMA and MWCNT/PMMA composites toward VOCs including dichloromethane, chloroform, and acetone were evaluated by Philip et al. [112]. The F-MWCNT/PMMA showed a significant sensing response (2–3 order of magnitude) and better reversibility to dichloromethane, chloroform, and acetone. The sensing mechanism of the sensor was explained based on the polymer swelling and polar interaction of the MWCNT surface with vapor molecules. 

### 4.4. FLN/Polymer Composite

Due to the extraordinary physical, chemical, and structural properties along with unique electronic properties of FLN, FLN/polymer composites received great attention in different areas, such as sensors, energy storage and conversion, drug delivery, and field emission devices [113]. Jiang et al. [114] investigated interfacial bonding between carbon fiber and epoxy modified with FLN nanoparticles by the addition of different FLN content (1–3 wt.%) into epoxy. By the incorporation of 2–3 wt.% FLN nanoparticles into the epoxy matrix, a remarkable increase in the filler/matrix bond strength and fracture toughness was obtained. The dispersion quality of FLNs in epoxy matrix was examined by TEM. The results indicated the relatively homogenous distribution of FLN nanoparticles into the epoxy matrix; however, some agglomeration was also observed. The improvement of interfacial bonding is attributed to the toughening effect of the epoxy matrix modified by FLN nanoparticles, which may dissipate deformation energy and reduce the stress concentration in the interface layer around the filler and prevent the de-bonding of filler and matrix.

**Figure 10:**
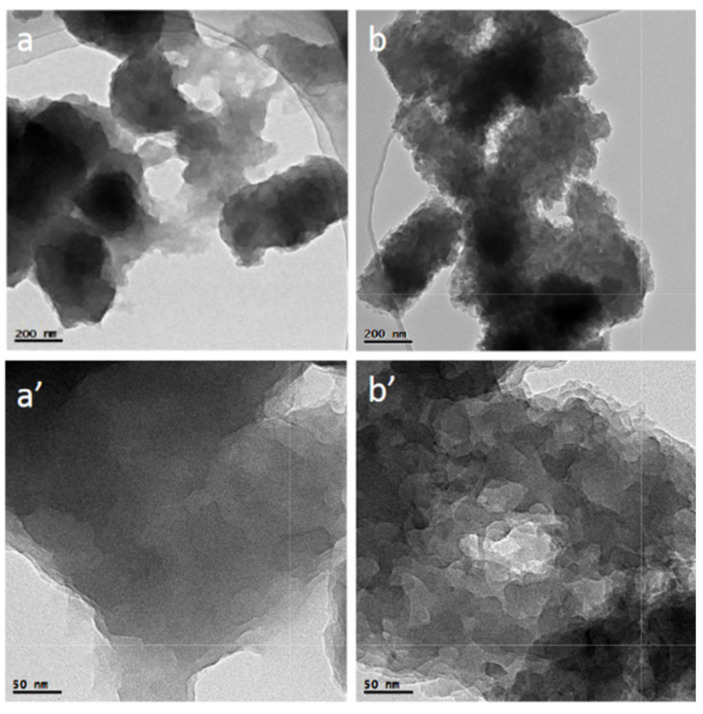
TEM images of (**a**,**a**’) PANI and (**b**,**b**’) FLN/PANI [118], reprinted with permission from Elsevier.

In another study, the effect of FLN dispersion on the properties of reinforced epoxy carbon filler composites was studied by Ogasawara et al. [115]. They studied the influence of FLN dispersion on the mechanical properties of epoxy. They reported a 60% enhancement in interlaminar fracture toughness by incorporating 0.1–1 wt.% FLN in the epoxy. Moreover, tension and compression strengths increased by 2–12% by dispersing 0.5 wt.% of FLN into the epoxy. Bronnikov et al. [116] investigated the effects of FLN loading on the electrical conductivity of a polyazomethine/FLN composite. It was observed that by the addition of small amount of FLN in the range of 0.25–0.5 wt.% into the polymer, the electrical conductivity of the composite is low due to the good dispersion of a small number of nanoparticles, which prevents the formation of the percolation network. While the addition of a larger amount of 2.5 wt.% FLN into the polymer increases the electrical conductivity of the composite because of percolation network formation. To investigate the role of surface modifications of FLN on the mechanical and electrical properties of FLN/polymer composites, Tayfun et al. [117] functionalized FLN using nitric acid and an amino functional silane coupling agent and then incorporated it in a thermoplastic polyurethane (TPU) matrix via the melt mixing method. The addition of 0.5 wt.% functionalized FLN into TPU caused an almost twofold increase in tensile strength and Young’s modulus values. The electrical properties of the FLN/TPU composite were also improved, which could be attributed to the better dispersion of FLN in the polymer matrix improvement of interactions between the FLN and TPU matrix. Cheng et al. [118] used FLN and PANI as hybrid nanoparticles for the incorporation into the polydivinyl benzene (PDVB) and studied their decoupling effect on electrical and thermal conductivity. The TEM images shown in Figure 10 demonstrated that the FLN/PANI hybrid (Figure 10b,b’) has the similar size to PANI (Figure 10a,a’) but a rougher surface, which may be related to the doped interaction between the imine groups of PANI and FLN.

Such doped interactions decrease the aggregation of PANI and enhance the electrical conductivity of related composites. The electrical conductivity was improved from 9 × 10^−10^ to 63.7 S·m^−1^ (more than 10 orders of magnitude) by incorporation of FLN/PANI hybrids into PDVB, while the thermal conductivity was extremely reduced resulting in effectively decoupling thermal/electrical conductivity. The electrical conductivity and mechanical properties of several nanocomposite materials based on FLN/polymer are compared in Table 6.

However, there is no report on an FLN/polymer composite for chemiresistive sensor applications, which may be due to the lack of percolation pathways because of their spherical structure. The low conductivity of this material compared to CNT or G-derivatives necessitate a higher loading range of this nanofiller into the polymer matrix, which suffers from improper dispersion and leads to high costs [119].

### 4.5. Carbon Black/Polymer Composite

The incorporation of CB into the polymer matrix creates the interface area between soft and solid phases. This feature benefits the prevention of permanent electrostatic discharge and the prevention of explosion, which is useful for the polymer composite applications [120]. Lohar et al. [121] fabricated a nanocomposite using polypropylene (PP) as polymer matrix, acrylonitrile-butadiene styrene (ABS) as rubber phase copolymer, and CB as nanofiller. They added different contents of CB (2.5–10 wt.%) in 80/20 (wt./wt.) PP/ABS blends and investigated the influence of CB on the mechanical behavior of polymer blends. The addition of 2.5 wt.% CB exhibited a 12% enhancement in tensile properties. Furthermore, 5 wt.% of CB addition in polymer blends of PP/ABS showed a two times improvement in impact strength compared to 80/20 (wt./wt.) neat PP/ABS blends. Kanbur [122] prepared the composites based on PP and different content of CB (1–30 wt.%) and studied the effect of CB content on the mechanical and electrical properties of related composites. The SEM results revealed that, by increasing the CB content from 5 to 30 wt.% in the PP matrix, the dispersion of CB in PP became more difficult. Upon the addition of 2 wt.% CB into PP, the electrical conductivity increased from 10^−14^ S·m^−1^ (neat PP) to 10^−4^ S·m^−1^ due to the extension of CB clusters into the polymer matrix. The composite containing a lower ratio of CB showed more flexibility than the sample containing a higher content of CB (>10 wt.%). After percolation the threshold point, the rapid increase in electrical conductivity was observed due to the formation of conductive layers by CB fillers.

Increasing filler content after the threshold point exhibited a negative effect on percent deformation at break. To reduce the filler aggregation and homogeneous dispersion in the polymer matrix, Liang et al. [123] modified the CB with MMA monomer (MCB) and synthesized the MCB/PMMA composites with different additive amounts of MCB (0.1–0.7 wt.%) using the in situ suspension polarization method. The MCB/PMMA composite with 0.5 wt.% additive amount of MCB demonstrated outstanding thermal and mechanical properties. The values of the electrical and mechanical properties are not reported in this manuscript. In Table 7, the electrical and mechanical properties of nanocomposites based on CB/polymers are given.

CB/polymer composites have been actively explored as sensing materials in chemiresistive sensors. Hopkins and Lewis [124] fabricated 20 arrays of CB/polymer composites using 10 different polymers and used them for the detection of the nerve agent simulants dimethylmethylphosphonate (DMMP) and diisopropylmethylphosphonate (DIMP). Developed arrays could easily detect and differentiate DMMP from the signatures of the other test analytes in the presence of backgrounds of potential interferences even at the very low concentration of DMMP. Later, Sisk and Lewis [125] prepared various composites filled with low mass fractions of CB (1–12 wt.%) and investigated their chemiresistive behavior towards 16 different analyte vapors. The low-mass-fraction CB/polymer composites were generally more sensitive and often afforded greater signal-to-noise ratios as compared to high-mass-fraction CB/polymer analogues. However, a lack of linearity in response vs. analyte concentration and less reproducibility tempered their advantages. Mallay et al. [126] fabricated the chemiresistive sensor based on 27 wt.% CB and 73 wt.% poly ((2, 5-dithienyl- 3,4-(1,8-naphthylene) cyclopentadienone)-co-4,7-bis(3-hexylthiophen-2-yl) benzo[c][1,2,5]thiadiazole (poly (DTCPA-co-BHTBT)) and used for the detection of VOCs including toluene, acetone, carbon tetrachloride, and cyclohexane. The proposed sensor exhibited higher sensitivity, selectivity, and reproducibility to toluene in a wide concentration range of 150–3000 ppm with LOD of 15 ± 10 ppm. Dispersion interaction of poly (DTCPA-co-BHTBT) with toluene is proposed to be the reason for the selective response towards toluene. To understand the physical and chemical mechanisms of CB/polymer composites as chemiresistive sensors, a mathematical model was developed by Lei et al. [127]. They represented 64 chemiresistive sensors with different CB concentrations (8–60 vol%) by repositioning a thin film of CB/polymer composite onto platinum electrodes on a silicon chip and used them for the detection of toluene and trichloroethylene. By using the designed model, the sensor responses for the given vapor pressures can be predicted. Moreover, the analyte vapor concentrations can be estimated based on the responses of the sensor. The sensing mechanism of CB/polymer composites has been assumed based on the adsorption and desorption of analyte on composite, which changes the connectivity pathways of CBs. The chemiresistive behavior of some CB/polymer composites is summarized in Table 8.

## 5. Conclusions and Future Outlooks

Carbon-based nanofiller/polymer composites are promising sensing materials for chemiresistors when they fulfill the requirements of homogeneous distribution of nanofiller into the polymer to allow a reproducible design of a percolation pathway. Thus, there have been successful attempts to develop nanocarbon-based polymer composites as sensitive films in chemiresistive sensors by choosing the right polymer–nanofiller pair, controlling of the film thickness, modifying fillers with functional groups for the improvement of the physical, chemical, and mechanical properties of the nanocomposite, and variations in the synthesis methods. The review summarized different synthesizing strategies for polymer nanocomposites. Among them, the solution mixing and in situ polymerization methods were more successful because they provide the most homogeneous distribution of fillers into the matrix and better mechanical and electrical properties. The different carbon nanofillers, such as G, GNR, CNT, FLNs, and CB, with their influence on the electrical, mechanical, and sensing properties of nanocomposites were investigated. It is noticeable that the morphology of nanocomposites and the dispersion of carbon nanofillers have an important impact on the sensitivity performance of related nanocomposites. Among the introduced nanofillers, the addition of a small amount of CNT as 1D and G-related derivatives as 2D nanomaterials in the polymer matrix demonstrated significant enhancement in the electrical conductivity and tensile properties of the final composites. The compatibility and better dispersibility of CNT and G-related nanomaterials with polymers provide a strong interfacial interaction between matrix and carbon fillers, resulting in better sensing performance compared to the composite material formed by spherical fillers such as FLNs and CB. In addition, the higher aspect ratio of G and CNTs can decrease the demands for the filler content to establish the percolation network [128,129]. A deep understanding of the sensing mechanisms, interaction sites (polymer or filler) as well as the type of interaction (hydrogen bonding, van der Waals, etc.) have the key roles to improving the sensing performance of the nanocarbon/polymer composites. However, there are still numerous challenges to design polymer nanocomposites for high-performance chemiresistive sensors. To further progress, intensive research on using functionalized fillers as well as using hybrid carbon-based fillers to tune the percolation pathway in the composite would be beneficial. Furthermore, the chemiresistive potential of GNRs/polymer can be studied intensively, because it has not been investigated even if the GNRs/polymer can offer a large potential for chemiresistive devices due to its excellent structural, chemical, and physical properties. Finally, these nanocarbons-based polymer composite chemiresistors have been mostly studied for the detection of gas and vapor samples, and there are only a few reports for liquid samples [130].

## Figures and Tables

**Table 1:** Electrical conductivity and mechanical properties of G, GO, rGO/polymer nanocomposites.

Polymer	Filler	Filler Content (wt.%)	Method	Conductivity (S·m^−1^)	Tensile Strength (MPa)	Young’s Modul (GPa)	Elastic Modul (%)	Ref.
PMMA	rGO	0.0 0.5–2 1–2 1–2	In situ polymerization Bulk polymerization Sheet casting	– 3.8 × 10^−4^–9.9 × 10^−3^ 4.1 × 10^−4^–0.018 9.5 × 10^−3^–0.17	24.2 23.0–14.2 26.0–13.0 28.0–26.0	0.75 0.86–0.85 0.77–0.89 0.96–1.05	2.8 2.6–1.5 2.2–1.5 2.2–1.7	[66]
PC	GO	1.09 (vol.%)	Solution mixing	0.041	n.a. *	n.a.	n.a.	[76]
PVA	rGO	4–14	Solution mixing	6.04 × 10^–3^–5.92	n.a.	n.a.	n.a.	[77]
PS	rGO	0.2–0.9 (vol.%)	Self-assembly	7 × 10^−7^–0.02	n.a.	n.a.	n.a.	[78]
PI	rGO r-I-Ph-GO	0 0.5–2 0.5–2	In situ polymerization	2.4 × 10^−13^ 2.1 × 10^−11^ –8.5 × 10^−9^ 1.4 × 10^−10^–0.092	~75.7 ~91–67 ~123–98	~2.5 ~4.5–5.6 ~6.8–9.6	~12 ~9.9–3.2 ~6.5–4.0	[79]
PS	G	0.11–0.69 (vol.%)	Solution mixing	6.7 × 10^−14 –^0.15	n.a.	n.a.	n.a.	[80]
PS	G	0.1 (vol.%)	Solution mixing	13.84	n.a.	n.a.	n.a.	[81]
PVA	CNT +GO	2–3	Solution mixing	n.a.	~6.5–8.5	~4–5	n.a.	[83]

* Not available.

**Table 2:** G, GO, rGO/polymer nanocomposite films as chemiresistive sensors.

Polymer	Filler	Filler Content (wt.%)	Analyte	Analyte Concentration (ppm)	Sensitivity (%)	LOD (ppm)	Ref.
PANI	GO	0.5	NH_3_	100	31.8	0.05	[67]
PEDOT: PSS	G	0 2.33	NH_3_	5–50, 500, 1000 5–50, 500, 1000	0.9–3.7, 4.4, 6.9 1.2–5.5, 9.6, 18.9	<10	[85]
PPy	rGO	5	NH_3_	33.2	~7	n.a. *	[86]
PANI	G	n.a.	NH_3_	20, 100	3.65, 11.33	1	[87]
PANI	GO	n.a.	Methanol Ethanol Propanol	100–500	20.9–37 3.77 3.10	n.a.	[88]
PEDOT	rGO	n.a.	NO_2_	2	20	n.a.	[89]
PMMA	G	0.01 g	Formaldehyde Methanol Acetone Tetrahydrofurane	2	10.4 2.0 1.3 1.1	0.01 - - -	[90]
PEDOT	PIL-rGO	n.a.	Methanol Benzene Chloroform Tetrahydrofuran	0–90	~3.0–4.0 ~2.2–3.5 ~2.0–2.5 ~1.5–2.0	1	[91]
PEDOT	rGO	n.a.	NO_2_	100	14–15	-	[92]
CMC	G	n.a.	Organic solvents Saline solution Polymer solution PVA solution	n.a.	n.a. ~0–100 ~0–25 ~0–20	n.a.	[93]

* Not available.

**Table 3:** Electrical conductivity and mechanical properties of GNRs/polymer nanocomposites.

Polymer	Filler	Filler Content (wt.%)	Method	Conductivity (S·m^−1^)	Tensile Strength (MPa)	Young’s Modul (GPa)	Elastic Modul (%)	Ref.
Epoxy	GNRs	~0.3	Solution mixing	n.a. *	n.a.	n.a.	n.a.	[28]
PVA	GNRs	0–2	Solution mixing	n.a.	18.2–33.8	0.070–1.164	n.a.	[29]
P(MMA-BA-HEMA)	GNRs	0.2–3	In situ polymerization	n.a.	n.a.	~ 1 × 10 ^−5^ – 7 × 10 ^−4^	n.a.	[30]
PANI	GNRs	n.a.	In situ polymerization	n.a.	n.a.	n.a.	n.a.	[94]
PVAM	EF-GNRs	0.15	Solution mixing	n.a.	n.a.	n.a.	14% > neat PVAM	[95]

* Not available.

**Table 4:** Electrical conductivity and mechanical properties of CNTs/polymer nanocomposites.

Polymer	Filler	Filler Content (wt.%)	Method	Conductivity (S·m^−1^)	Tensile Strength (MPa)	Young’s Modul (GPa)	Elastic Modul (%)	Ref.
SBR	MWCNTs	10	Solution mixing	n.a. *	~7.5	n.a.	n.a.	[99]
iPP	SWCNTs	0–0.75	Solution mixing	n.a.	30.8–35.5	0.855–1.187	n.a.	[100]
PS	MWCNT/copolymer MWCNTs SWCNTs DWCNTs	5	Electro spinning	0.0053 – 0.037 0.0050	0.61 0.18 0.22 0.78	0.0163 0.007 0.0104 0.0234	19.4 10.8 8.6 12.3	[101]
PVAc	SWCNTs	0–5	Emulsion mixing	n.a.	n.a.	n.a.	n.a.	[102]
PU	- AP-SWCNTs EST-SWCNTs	n.a.	Electro spinning	n.a.	7.02 10.26 14.32	n.a.	n.a.	[103]
PMMA	SWCNTs SOCl_2_- SWCNTs	10 13	Solution mixing	1700 10 ^4^	(30–7.5) ** (370-330) **	** (0.4-0.2) ** (0.5-0.6)	n.a.	[104]
PMMA	SOCl_2_- SWCNTs	0.1–0.5	Solution mixing	0.35–47	n.a.	n.a.	n.a.	[105]
PVC	PBMA- MWCNTs	0–0.5	ATRP	n.a.	30.5–52.5	1.35–1.61	n.a.	[106]

* Not available ** (filler content: 0.1–1%).

**Table 5:** CNTs/polymer nanocomposite films as chemiresistive sensors.

Polymer	Filler	Filler Content (wt.%)	Analyte	Analyte Concentration (ppm)	Sensitivity (%)	LOD (ppm)	Ref.
PANI -	C-MWCNTs	n.a. *	NH_3_	2–10	15.5–32.0 2.58–7.20	n.a.	[84]
PANI	MWCNTS	33 **	NH_3_	0.2–15	~0.01–0.3	0.2	[108]
A: PEDOT: PSS B: PANI	MWCNTS	n.a.	NH_3_	20	A: ~0–15 B: ~0–12	n.a.	[109]
A: p-PANI B: n-PANI	MWCNTs	n.a.	NO_2_, NH_3_	50	A: 65.9, 0.975 B: 0.30, 276.3	0.0167, 0.0064	[110]
PANI	MWCNTs	25	AHV	200–1000	1–25	n.a.	[111]
PMMA	F-MWCNT MWCNT	25	VOCs	n.a.	1.04–809 2.243–9.94	n.a.	[112]

* Not available. ** PANI content. A and B represent the polymer type and the related sensitivity.

**Table 6:** Electrical conductivity and mechanical properties of FLN/polymer nanocomposites.

Polymer	Filler	Filler Content (wt.%)	Method	Conductivity (S·m^−1^)	Tensile Strength (MPa)	Young’s Modul (GPa)	Elastic Modul (%)	Ref.
Epoxy	FLN	1–3	Solution mixing	n.a. *	~90–92	~2.8–3	n.a.	[114]
Epoxy	FLN	0.1–1	Solution mixing	n.a.	~82–86	n.a.	n.a.	[115]
Polyazomethine	FLN	0.25–2.5	Solution mixing	(4 × 10^−4^–1.6 × 10^−3^) **	n.a.	n.a.	n.a.	[116]
TPU	FLN	0.5–2	Hot melt extrusion	Modified	~38–30	~65–54	~***(574–347)	[117]
PVDB	FLN- PANI	1.05 vol.%	Solution mixing	63.7	n.a.	n.a.	n.a.	[118]

* Not available. **At 170 °C. *** Elongation at break.

**Table 7:** Electrical conductivity and mechanical properties of CB/polymer nanocomposites.

Polymer	Filler	Filler Content (wt.%)	Method	Conductivity (S·m^−1^)	Tensile Strength (MPa)	Young’s Modul (GPa)	Elastic Modul (%)	Ref.
PP	CB	0–2.5 0–5	Hot melt extrusion	n.a *	~27–33.5 ~27–31	n.a.	n.a.	[121]
PP	CB	0–5	Melt mixing	10^−14^–10^−4^	~(30–37) **	~(0.55–1.10) **	(900–8) **	[122]
PMMA	MCB	0.1–0.7	In situ polymerization	n.a.	n.a. ***	n.a. ***	n.a. ***	[123]

* Not available. ** Injection molding, for 1–30% addition of CB. *** Significant improvement in mechanical properties.

**Table 8:** CB/polymer nanocomposite films as chemiresistive sensors.

Polymer	Filler	Filler Content (wt.%)	Analyte	Analyte Concentration (ppm)	Sensitivity (%)	LOD (ppm)	Ref.
A: ^1^ PEO B: ^2^ PEVA C: ^3^ PCL D: ^4^ PBS	CB	^5^ n.a.	DMMP in air	(0.0017, 0.0054, 0.013) *	A:0.0869, 0.0964, 0.0834 B: 0.188, 0.209, 0.170 C: 0.577, 0.531, 0.612 D: 0.146, 0.163, 0.133	A: 0.14 B: 0.050 C: 0.059 D: 0.19	[124]
			DIMP in air		n.a.	A: 0.19 B: 0.074 C: 0.049 D: n.a.	
A: ^6^ PEP B: ^7^ PVS	CB	12, 40 1, 40	Isooctane		A: 0–7.5, 0–0.76 B: 0–8, 0–0.26	n.a.	[125]
			^8^ THF		A: 0–22.5, 0–0.6 B: 0–6, 0–0.15		
			Chloroform		A: 0–14, 0–0.7 B: 0–7.8, 0–0.28		
P(DTCPA-co-BHTBT)	CB		Toluene	150–3000	0.39–2.02	15 ± 10	[126]
Polyisobutylene	CB	8–60 vol.%	Toluene Trichloro ethylene	n.a.	n.a. **	n.a.	[127]

^1^ Poly (ethylene oxide);^2^ poly (ethylene-co-vinyl acetate), 45% vinyl acetate; ^3^ poly (caprolactone); ^4^ poly (butadiene-co-styrene), 72% butadiene; ^5^ not available; ^6^ poly (ethylene-co-propylene); ^7^ poly (vinyl stearate); ^8^ tetrahydrofuran. * Partial pressure/vapor pressure (P/P0) of analyte at room temperature. ** The sensor response decreased with rising CB amounts. A, B, C and D represent the polymer type and the related sensitivity.
